# Quantification of Sonicated Implants from Patients with Osteoarticular Implant Infections

**DOI:** 10.3390/antibiotics15030258

**Published:** 2026-03-02

**Authors:** L. Trallero-Calvo, A. Auñon, A. Blanco, J. Garcia-Cañete, R. Parrón, J. Esteban, L. Salar Vidal

**Affiliations:** 1 Department of Clinical Microbiology, IIS-Fundación Jiménez Díaz, Universidad Autónoma de Madrid, Av. Reyes Católicos 2, 28040 Madrid, Spainllanos.salar@quironsalud.es (L.S.V.); 2 Department of Orthopaedic Surgery, Fundacion Jimenez Diaz University Hospital, Universidad Autónoma de Madrid, 28040 Madrid, Spain; alvaro.aunon@fjd.es (A.A.); ablancog@fjd.es (A.B.); raul.parron@quironsalud.es (R.P.); 3 CIBERINFEC-CIBER de Enfermedades Infecciosas, 28029 Madrid, Spain; jcanete@fjd.es; 4 Department of Internal Medicine-Emergencies, Fundacion Jimenez Diaz University Hospital, Universidad Autónoma de Madrid, 28040 Madrid, Spain

**Keywords:** sonication, quantification, CFU, prosthetic joint infection, culture

## Abstract

**Background:** Sonication of retrieved implants has emerged as a valuable diagnostic adjunct for Prosthetic Joint Infection (PJI), particularly in chronic infections or cases with prior antibiotic exposure. Quantitative culture of sonication fluid has been proposed to differentiate contamination from true infection; however, the diagnostic thresholds remain inconsistent across studies and may be influenced by methodological variability. **Objectives:** We aimed to evaluate bacterial counts obtained from the routine sonication of osteoarticular implants and assess their diagnostic performance across different infection types. **Methods:** A retrospective study was conducted (2011–2023) at a tertiary hospital. Implants from patients with PJI or Fracture-Related Infection (FRI), classified according to international criteria, were processed using a standardized sonication protocol, including centrifugation and inoculation onto multiple culture media. Quantitative results were expressed as CFU/mL. Bacterial counts were compared across infection types (acute PJI, chronic PJI, FRI), microbial characteristics, infection pattern, and affected joint using non-parametric tests. **Results:** A total of 457 sonicated implants were analyzed, including 316 PJI samples (26.3% acute; 73.7% chronic) and 141 FRI samples. The median bacterial count was 40,000 CFU/mL (IQR 1000–100,000). No significant differences were found between prosthetic and osteosynthesis implants. Polymicrobial infections showed significantly higher counts than monomicrobial infections (*p* < 0.005). No significant differences were observed according to Gram stain or joint site. Acute PJI tended to show higher bacterial burdens than chronic PJI, although not significantly (*p* = 0.052). **Conclusions:** Quantitative sonication yields substantial variability in bacterial loads, with higher counts in polymicrobial infections and a trend toward increased counts in acute PJI. A threshold of ≥1000 CFU/mL appeared to be clinically meaningful within our protocol. These findings support the diagnostic utility of quantitative sonication and underscore the need for protocol-specific thresholds.

## 1. Introduction

Periprosthetic joint infections pose a diagnostic challenge, as diagnosis is typically established through a combination of clinical, laboratory, and microbiological criteria, according to most guidelines [1,2]. Among the latter, synovial fluid and periprosthetic tissue cultures obtained during surgery are considered the gold standard for diagnosing prosthetic joint infection and identifying the etiology of the disease [1,3]. Over the last few decades, the development of new techniques and protocols has enabled the optimization of these culture methods, leading to improved diagnostic performance [1].

Since 2007, implant sonication has been shown to significantly enhance the sensitivity and specificity of culture [4,5,6,7], particularly for chronic periprosthetic infections, where low-virulence organisms are predominant, and in cases where prior antibiotic treatment has been administered. Although suspending antibiotic therapy before surgery is the most widely recommended practice in these cases, when such discontinuation is not feasible, sonication allows for achieving higher sensitivity compared to conventional methods [8]. Moreover, in these cases, biofilm formation, which is the essential pathogenic mechanism, can hinder bacterial recovery and, therefore, microbiological diagnosis. The ultrasonic treatment used in sonication has demonstrated favourable results, allowing for the recovery of sessile bacteria in these cases [9,10]. Because of these advantages, sonication is increasingly used in many laboratories, while its universal use has not yet been achieved [11].

In addition to qualitative identification, the quantification of bacterial counts in colony-forming units per millilitre (CFU/mL) from sonication fluid has been considered an important criterion for distinguishing contamination from true infection. Thresholds of ≥50 CFU/mL have been proposed to differentiate contamination from true periprosthetic infection [2]. However, factors included in the different sonication protocols, such as sample volume, centrifugation time and/or force applied, as well as the type of infection (monomicrobial or polymicrobial), may influence these counts, potentially altering their diagnostic value. Moreover, most studies obtained results through experimental study, and there are only a few studies that examined the use of sonication as a diagnostic tool in a routine setting [12,13].

This study aimed to assess the bacterial counts obtained from the sonication of osteoarticular implants and to analyze their diagnostic value across different types of joint infections in a routine setting.

## 2. Results

A total of 457 sonication fluid samples from patients with hip or knee prosthetic joint infections were analyzed. Of these, 316 were from patients diagnosed with PJI, of whom 26.3% (83/316) were classified as acute PJI (both post-surgical and late-acute) and 73.7% (233/316) as chronic PJI. Additionally, 141 samples from patients with FRI were included in this study. The mean age of the patients was similar across the three groups, with no clinically relevant differences observed (71.5 years in APJI, 71.2 years in CPJI, and 62.4 years in FRI). Sex distribution was balanced in all groups.

**Figure 1:**
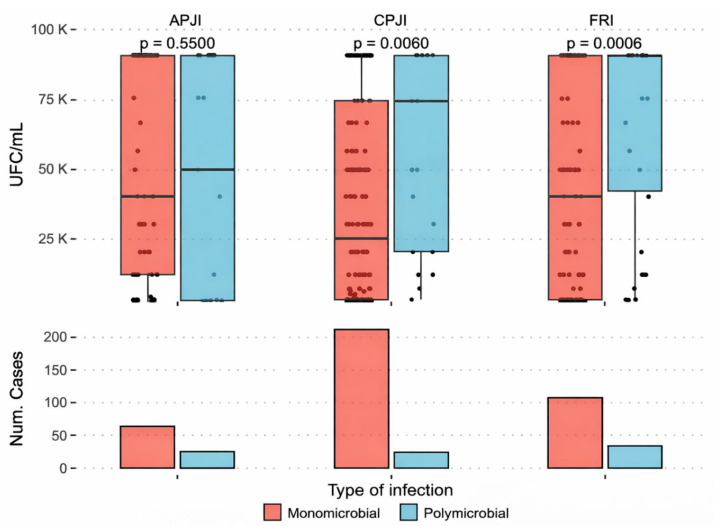
Bacterial count (CFU/mL) according to infection category. APJI: Acute Prosthetic Joint Infection; CPJI: Chronic Prosthetic Joint Infection; FRI: Fracture-Related Infection.

When all sonication fluid samples were pooled (APJI, CPJI, and FRI), the median bacterial count in sonication fluid was 40,000 CFU/mL (IQR 1000–100,000 CFU/mL). No statistically significant differences were observed between implant types (prosthesis vs. osteosynthesis, *p* = 0.218). Higher bacterial counts were observed in samples from acute PJI compared to chronic PJI, although this difference did not reach statistical significance (*p* = 0.052) (Figure 1).

**Figure 2:**
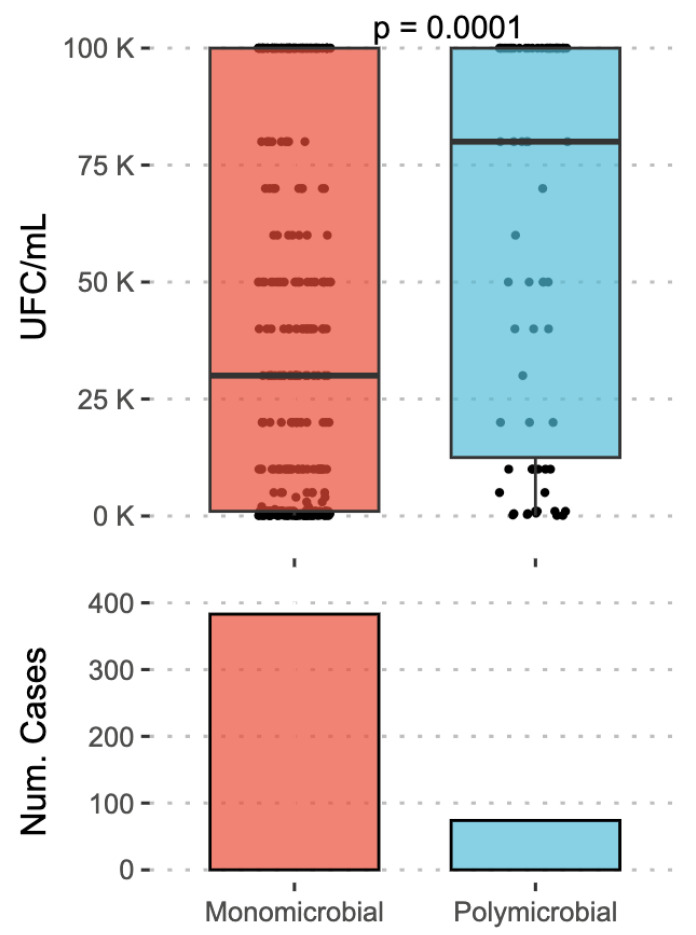
Bacterial count (CFU/mL) according to infection type.

Most infections were monomicrobial, whereas 16.2% were polymicrobial. Significantly higher bacterial counts were observed for polymicrobial infections compared to monomicrobial infections (*p* < 0.005) (Figure 2). Regarding monomicrobial infections, no significant differences in bacterial counts were observed between Gram-positive and Gram-negative microorganisms (*p* = 0.416). Likewise, no differences were found in bacterial counts according to the affected joint (knee vs. hip) (*p* = 0.353).

In APJI, the most frequently isolated microorganism was *Staphylococcus aureus*, followed by *Enterococcus faecalis* and *Staphylococcus epidermidis*. Gram-negative bacilli. accounted for a substantial proportion of isolates, and most infections were monomicrobial. In CPJI, *Staphylococcus epidermidis* was the predominant pathogen, followed by *Staphylococcus aureus* and *Cutibacterium acnes*. Polymicrobial infections were more frequently observed in this group. In infections associated with FRI, *Staphylococcus aureus* was the most commonly isolated microorganism; however, no single pathogen predominated. This group exhibited high etiological diversity, including Gram-negative bacilli, anaerobic bacteria, and fungal isolates, reflecting the greater microbiological complexity of infections related to the osteosynthesis material.

## 3. Discussion

In our study, bacterial counts of ≥1000 CFU/mL in sonicated samples were consistently observed among confirmed PJI when interpreted in correlation with clinical criteria, as well as other microbiological parameters such as sample type and/or number of specimens [1]. Given the absence of an aseptic control group, this threshold should be interpreted as an observation within the context of our cohort and sonication protocol rather than as a validated diagnostic cutoff. In fact, in a previous study from our group [14], we detected colony counts higher than 1000 CFU/mL among patients without PJI (negative controls), which were considered contaminants. These isolates mainly consisted of non-fermenting Gram-negative rods of environmental origin, highlighting the importance of clinical and microbiological correlation when interpreting the quantitative sonication results.

Our findings differ from previous studies, such as Álvarez-Otero et al. [15], who established a cutoff of 20 CFU/10 mL, but are more consistent with those of Rothenberg et al. [4], who proposed a cutoff of 5 CFU/plate as suggestive of infection. These differences are probably attributable not only to methodological variations among sonication protocols, but also to conceptual differences in study design. In our protocol, the limit for detection of a positive result (minimum 1 CFU/plate) corresponds to 1000 CFU/mL in the original sonicate fluid, a breakpoint higher than that used in other studies and guidelines [2,8].

The higher bacterial recovery observed in acute PJI compared to chronic PJI could be explained by the role of biofilm. In chronic PJI, most bacteria are embedded in the extracellular matrix of the biofilm, which acts as a protective layer preventing bacterial release [9] and thereby reducing the bacterial count obtained. Moreover, in acute infections, there is a high bacterial load of planktonic organisms, and it is possible that some of these organisms can be attached to the implant before the sonication procedure is performed. This weak attachment of bacteria in these infections has been used as the basis of an alternative procedure using vortexing, which showed good results among acute infections [16]. Some protocols, including the original by Trampuz et al. [8] include vortexing as a procedure prior to sonication. It is likely that, during this step, weakly attached bacteria are dislodged, and ultrasonic treatment affects only those strongly. attached sessile organisms. These findings suggest that clinical guidelines should consider differentiating between acute and chronic infections when establishing a breakpoint for interpreting bacterial counts, as well as differences between protocols for the evaluation of quantification of the results.

Although previous studies have reported higher recovery rates for Gram-positive bacteria due to their greater resistance to sonication processing, no significant differences between Gram-positive and Gram-negative bacteria were observed in our study [17].

Regarding infection type, it is estimated that approximately 85% of prosthetic infections are monomicrobial [18]. Although evidence is limited, Tanz et al. documented that polymicrobial infections are associated with poorer outcomes. This may be explained by the fact that, in many of these cases, tissue quality is compromised, facilitating the entry of additional contiguous bacteria, which not only increases bacterial counts, but also leads to higher rates of therapeutic failure [19]. In our study, higher bacterial counts were observed in polymicrobial infections; a possible explanation is that hypoxic environments and poorer tissue conditions may promote the release of nutrients utilized by bacteria, as well as residual metabolites that enhance mutual bacterial growth, leading to higher bacterial proliferation.

Although our study did not identify significant differences in bacterial counts between prosthetic implants and osteosynthesis devices, the previous literature has reported distinct microbiological profiles according to the type of implant-associated infection. In addition to these microbiological differences, at least one study has also reported higher rates of prosthetic joint infection in patients with prior or concomitant osteosynthesis hardware, suggesting that the presence of such material may predispose to subsequent infection [20]. Furthermore, several studies [21,22] have described differing pathogen distributions in prosthetic joint infections versus Fracture-Related Infections, as well as a higher frequency of multidrug-resistant organisms in the latter. These observations highlight the importance of considering these clinical and microbiological differences in future studies to better inform antimicrobial treatment strategies.

Although implant sonication is not a novel technique and has been used for many years in the diagnosis of implant-associated infections, the innovative aspect of the present study lies in the application and evaluation of a standardized quantitative sonication protocol in a real-life clinical setting over a long study period. In contrast to the original protocol described by Trampuz et al. [8] and other commonly used methodologies, our protocol incorporates a centrifugation step to concentrate the sonication fluid prior to culture, allowing for quantitative assessment under routine diagnostic conditions.

Importantly, most previously published studies addressing quantitative thresholds were based on experimental designs or limited case series, whereas our work reflects daily clinical practice in a tertiary care hospital, including a large and heterogeneous cohort of prosthetic joint infections and Fracture-Related Infections. This pragmatic approach provides clinically applicable data on bacterial load distribution and supports the concept that quantitative cut-off values should be interpreted in the context of the specific sonication protocol used. Our findings emphasize the need for protocol-specific interpretation of quantitative sonication results in routine microbiology laboratories.

Recent studies have shown that the combined use of sonication and blood culture bottles for incubation of synovial fluids can increase sensitivity by up to 100%. However, these approaches raise concerns regarding specificity, as the inability to perform quantitative counts makes it difficult to establish the clinical relevance of the microorganism [23]. Similarly, molecular techniques, including multiplex PCR assays, provide rapid pathogen detection, but do not distinguish between viable and non-viable microorganisms and lack quantitative information, which can limit their clinical interpretation [24]. In this context, quantitative sonication represents a feasible diagnostic approach that can be easily implemented in routine clinical practice, offering additional information on bacterial burden when interpreted alongside clinical and microbiological criteria.

This study has several limitations. It was a retrospective analysis conducted at a single centre using a standardized sonication protocol during the entire period. Only hip and knee among all periprosthetic infections were evaluated, and no clinical data of the patients (especially previous antibiotic intake) were recorded. Importantly, all analyzed cases corresponded to confirmed implant-associated infections. Implants removed for non-infectious reasons (such as aseptic loosening, pain, or soft tissue irritation) were not included. As a result, only microbiologically positive sonication cases were analyzed, and aseptic revision cases were not available as a control group, which did not allow for comparison with microbiologically negative sonication results and limited our ability to validate sonication cut-off values. Species-level differentiation and individual bacterial counts were not assessed, and no species analysis was performed.

## 4. Materials and Methods

A retrospective study was conducted between January 2011 and October 2023 in a tertiary care hospital in the Community of Madrid. During this period, according to current international diagnostic criteria, all implants (prostheses, osteosynthesis materials) obtained from patients diagnosed with prosthetic joint infection (PJI) [2,25,26,27] or Fracture-Related Infection (FRI) [28] were included. Of interest, among these guidelines, only the EBJIS criteria include sonication and a tool for microbiological diagnosis [2].

During the study period, implants were sonicated according to a previously established procedure [29]. This procedure includes the use of centrifugation (20 min at 3000× *g*) for a 10× concentration of the sonicate fluid, and after this procedure, 10 μL of this concentrated sonicate were inoculated in 4 plates, Tryptic soy-5% sheep blood agar (TSS), Chocolate agar (CH), Schaedler-5% sheep blood agar (SCS), and McConkey agar (McC), all of them from Biomérieux (Marcy l’Etoile, France). All the media were incubated in different atmospheres (normal atmosphere for McC, 5% CO_2_-enriched atmosphere for TSS and CH, and anaerobic atmosphere for SCS) at 35–37 °C during a minimum of 7 days, which was extended to 14 days when infection was strongly suspected. Along with the implant samples, from three to six periprosthetic tissue samples were collected, and in cases of suspected PJI, synovial fluid was also obtained. Tissue samples were homogenized by grinding, and both tissue and synovial fluid samples were placed in culture and incubated under the same conditions as the sonicated implant samples.

Bacterial counts were registered as an average of those obtained in all the media, and they were expressed as colony-forming units per millilitre (CFU/mL). Bacterial count was adjusted to the original volume of sonicated fluid previous to centrifugation. Counts higher than 100,000 CFU/mL were recorded as 100,000 CFU/mL for statistical purposes. Among polymicrobial infections, only one count was recorded (that of the most abundant organisms).

Data were analyzed using non-parametric tests, as bacterial count did not follow a normal distribution. Comparisons of bacterial counts (CFU/mL) between groups were performed using the Mann–Whitney U test. Differences were assessed according to infection type (acute PJI, chronic PJI, or FRI), type of microorganism (Gram-positive vs. Gram-negative), infection pattern (monomicrobial vs. polymicrobial), and affected joint (hip vs. knee). Statistical significance was defined as *p* < 0.05. Data analysis was performed using R software (version 4.3).

This study was approved by the ERC of our hospital (reference EO053-21_FJD).

## 5. Conclusions

Significant variability in bacterial counts was observed across all sonication samples, independent of implant type or microbial species. Polymicrobial infections exhibited significantly higher counts than monomicrobial infections, and a trend toward elevated counts was noted in acute prosthetic joint infections. These findings clarify the diagnostic value of sonication combined with quantitative bacterial assessment for identifying true infections. These results may help guide clinical decisions and targeted antibiotic therapy.

Further multicentre studies are warranted to refine quantitative thresholds and better understand the clinical implications of polymicrobial infections.

## Data Availability

All data are included in the Supplementary Material. Any other data are available upon request.

## Supplementary Materials

The following supporting information can be downloaded at https://www.mdpi.com/article/10.3390/antibiotics15030258/s1.
